# Clinical significance of epithelial-mesenchymal transition

**DOI:** 10.1186/2001-1326-3-17

**Published:** 2014-07-02

**Authors:** Konrad Steinestel, Stefan Eder, Andres Jan Schrader, Julie Steinestel

**Affiliations:** 1 Bundeswehr Institute of Radiobiology, Neuherbergstrasse 11, Munich 80937, Germany; 2 Institute of Pathology and Molecular Pathology, Bundeswehrkrankenhaus Ulm, Oberer Eselsberg 40, Ulm 89081, Germany; 3 Department of Urology, Ulm University Medical Center, Prittwitzstrasse 43, Ulm 89075, Germany

**Keywords:** Epithelial-mesenchymal transition, Invasion, Metastasis, Prognosis, Therapy

## Abstract

The concept of epithelial-mesenchymal transition (EMT), a process where cells change their epithelial towards a mesenchymal phenotype, has gained overwhelming attention especially in the cancer research community. Thousands of scientific reports investigated changes in gene, mRNA and protein expression compatible with EMT and their possible correlation with tumor invasion, metastatic spread or patient prognosis; however, up to now, a proof of clinical significance of the concept is still missing. This review, with a main focus on the role of EMT in tumors, will summarize the basic molecular events underlying EMT including the signaling pathways capable of its induction as well as changes in EMT-associated protein expression and will very briefly touch the role of microRNAs in EMT. We then outline protein markers that are used most frequently for the assessment of EMT in research and diagnostic evaluation of tumor specimens and depict the link between EMT, a cancer stem cell (CSC) phenotype and resistance to conventional antineoplastic therapies. Furthermore, we evaluate a possible correlation between EMT marker expression and patient prognosis as well as current therapeutic concepts targeting the EMT process to slow down or prevent metastatic spread of malignant tumors.

## Introduction

Epithelial-mesenchymal transition (EMT) is a central element of embryonic development, wound healing and tumor cell migration, and has thus obtained much attention by the research community since Greenburg and Hay firstly described a mesenchymal-like transformation of epithelial cells when suspended in collagen gels [1]. Basically, the term describes a process in which cells lose epithelial and gain mesenchymal characteristics; this is accompanied by a loss of cell-cell cohesiveness, leading to enhanced migratory capacity [2]. Multiple genes as well as proteins that seem to play a central role in EMT have so far been identified and are either up- or downregulated during the process, thus serving as possible markers in the assessment of EMT. Since it seems to be a key element in wound healing and tumor cell migration, there is also great interest in EMT as a pharmaceutical target; recent publications even proposed vaccination against drivers of EMT as an immunotherapeutic approach against tumor progression [3].

However, since many studies on EMT are based on *in vitro* results and not all findings could be confirmed *in vivo*, the clinical significance of the concept remains unclear [4]. This review lays the main focus on EMT in tumor cells and aims at recapitulating what is known about the molecular basis of EMT. Furthermore, we will summarize current markers of EMT that are in clinical and/or diagnostic use and, finally, evaluate EMT from a translational point of view and in the context of clinical feasibility.

## Review

### The molecular basis of EMT

**Figure 1:**
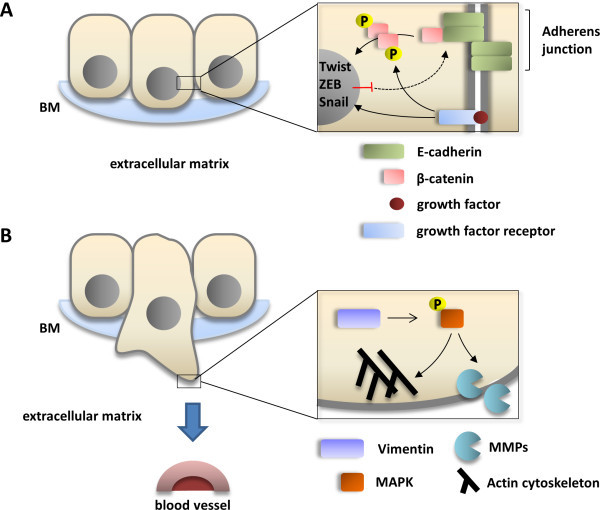
**Basic molecular changes underlying EMT. A**, Signaling along canonical TGF-β pathway activates EMT-promoting transcription factor (such as Twist, ZEB or Snail) to repress transcription of E-cadherin that initially forms the adherens junction (AJ) complex together with β-catenin. Extinction of E-cadherin from the AJ complex as well as concomitant phosphorylation via activated growth factor receptors lead to cytoplasmic accumulation and nuclear translocation of β-catenin, where it acts as a transcription factor for migration-associated genes. **B**, Enhanced expression of Vimentin in migrating tumor cells protects phosphorylated MAPK from cytoplasmic phosphatases, thus ensuring signaling activity along the EGFR/MAPK axis. This supports pro-migratory effects on the cytoskeleton (such as Rac-mediated actin polymerization) and secretion of lytic matrix metalloproteinases that cleave the surrounding extracellular matrix to allow for cell migration.

Basically, EMT stands for a loss of epithelial and a gain of mesenchymal cellular characteristics that enhance migration and invasion by the cell [5]. This process includes loss of cell cohesiveness as well as fundamental reorganization of the cytoskeleton inducing a switch from apical-basal to front-rear polarity, and may furthermore be associated with the acquisition of invasive properties through the secretion of lytic proteases as well as resistance to senescence and apoptosis [6]. EMT is under tight control of multiple regulatory pathways; first and foremost, transforming growth factor β (TGF-β) signaling activity is enhanced in many physiological and pathological conditions in which EMT is observed, such as organogenesis, inflammation and tumor invasion [7,8]. In canonical TGF-β signaling, binding of TGF-β to its cell surface receptors (type I-III) activates complex formation of Smad family transcription factors, which translocate to the nucleus and cooperate with transcription factors from the Snail and Twist family, so-called “EMT master genes” [9,10]. Non-Smad signaling molecules downstream TFG-β and supportive of EMT include activated Rho-like GTPases, Phosphatidylinositol-3-kinase (PI3K) and mitogen-associated protein kinase (MAPK; the various signaling pathways mediating TGF-β signaling in EMT are excellently reviewed in [6]). Taken together, these effectors mediate transcriptional repression of genes that are involved in cell polarity and cell-cell adhesion, such as RhoA and E-cadherin (Figure 1A) [11,12]. The latter is mediated by the recruitment of histone deacetylases (HDACs) and other repressors to E-box elements in the E-cadherin promoter, leading to chromatin condensation and transcriptional repression [13]. At the same time, the expression of N-cadherin, another member of the cadherin family that allows for enhanced adhesion between mesenchymal cells, is upregulated; this balanced change in cadherin expression has thus been designated “cadherin switch” and is regarded a hallmark of EMT [14]. Not only the expression, but also specific membraneous targeting of E-cadherin is repressed in EMT via loss of the epithelial-specific intermediate filament keratin; therefore, loss of keratin immunostaining is widely regarded as a marker for ongoing EMT [15,16]. Further mechanisms that lead to degradation of cell-cell junctions include a repression of claudin and occludin expression, while zonula occludens 1 (ZO-1) is subsequently lost in a post-transcriptional manner [17-19]. This repression is maintained throughout further progression of EMT [20]. Since protein complexes (such as partitioning defective – PAR) that define the apical compartment of the cell are normally associated with intercellular junctions, degradation of the junctions also weakens the apical-basal polarity cellular phenotype [6]. Moreover, the TGF-β-facilitated signaling along the MAPK axis exerts pro-proliferative and anti-apoptotic effects on the cell, while Ras/MAPK activity alone – without TGF-β induction - has also been linked to enhanced EMT [21-23]. After losing cohesiveness due to degradation of cell-cell junction complexes, mesenchymal-like tumor cells are able to invade through the basement membrane into underlying tissue by the secretion of lytic enzymes such as matrix metalloproteinases (MMPs) and MAPK-mediated reorganization of the actin cytoskeleton which is enhanced by the expression of Vimentin (Figure 1B) [24]. In detail, migration and invasion of moving cells is facilitated by specialized cellular protrusions, such as filopodia, lamellipodia and invadopodia. While filopodia, consisting of actin filaments arranged in a parallel fashion, seem to sense changes to the cellular microenvironment and act as a “guide” through the surrounding matrix, lamellipodia are built upon a branched actin network and allow for actin-myosin interactions as a prerequisite for cellular movement [25,26]. Both filopodia and lamellipodia have been linked to an EMT-like phenotype in migrating tumor cells [27,28]. Invadopodia are closely related to lamellipodia in a sense that they also consist of a branched network of actin filaments, but have the ability to degrade the extracellular matrix (ECM) through the secretion of lytic proteases, such as MMP-1, MMP-7 and MMP-9 (Figure 1B) [26]. Invadopodia formation has been linked to activity of the EMT transcription factor Twist1 cancer, and own results showed high expression of invadopodia-associated proteins, such as Cortactin and Abelson interactor 1 (Abi1), in a colorectal carcinoma cell line with an EMT-like phenotype shown by loss of E-cadherin [29,30]. Accordingly, TGF-β signaling activates small GTPases that enhance local reorganization of the actin cytoskeleton as a prerequisite for lamellipodia and filopodia formation, such as Rho, Rac and Cdc42 [31]. Vimentin, which is frequently upregulated in cells with an EMT-like phenotype, is then required for the further maturation of invadopodia [32]. Besides clearing the way for migrating tumor cells, MMPs that are released during tumor cell invasion are themselves further fueling the EMT process; the same effect is achieved via liberated TGF-β from the ECM [33-35]. In a mouse model of gastric cancer, it could be shown that EMT cooperates with MMP activity to gain access to lymph vessels and to spread distant metastases [36]. Accordingly, blood and lymph vessel infiltration by triple-negative breast cancer cells is associated with the expression of EMT transcription factor Zeb1 in surrounding stroma [37]. Alterations in MMP expression are linked to changes in the integrin repertoire with downregulation of some (epithelial) and upregulation of other integrins that facilitate interaction with extracellular matrix components such as collagen [6]. Targeting transmembrane proteins - like E-cadherin - or increasing the levels of intracellular reactive oxygen species via enhanced activation of Rac1b are further mechanisms of MMP-induced EMT [38,39].

Upon arrival at the site of metastasis, it seems a prerequisite for metastatic colonization that tumor cells undergo a partial reversal of the EMT, the so-called “mesenchymal-epithelial transition” (MET) [40,41]. During that process, tumor cells regain the expression of epithelial markers, such as E-cadherin, while the expression of EMT-associated transcription factors, such as Twist1, is repressed [41]. Thus, EMT can be seen as a reversible and transient process that enables epithelial tumor cells to gain access to the vasculature, allowing for the formation of distant metastasis.

Besides TGF-β, other signaling pathways have also been implied in the activation of EMT; for example, hypoxia-inducible factor (HIF) contributes to EMT in tissue fibrosis and cancer cell invasion by modulating the activity of pro-EMT transcription factors Notch and β-catenin [42-44]. HIF1α induces Twist and Snail expression in endothelial as well as ovarian carcinoma cells [45-47]. Additionally, activation of several receptor tyrosine kinases (RTKs) may result in induction of EMT; in these scenarios, growth factor binding to RTKs as well as activating mutations in oncogenes downstream of the receptors leads to enhanced signaling along the Ras/MAPK or Akt/mTOR axis, resulting in upregulation of Snail expression [6]. Finally, it has been shown that enhanced wnt signaling activity as well as an upregulation of chemokine receptors (such as CXCR-1) also support the process of EMT [48,49]. Here, wnt signaling leads to an inhibition of glycogen synthase kinase 3β (GSK3β)-mediated phosphorylation of β-catenin; the resulting decrease in proteosomal degradation and cytoplasmic accumulation of β-catenin supports its translocation to the nucleus, where it acts as a transcriptional co-activator of EMT-associated gene expression [50].

In the recent years, the role of small, non-coding RNAs in the EMT process has also been further elucidated. Methylation-depedent expression changes in levels of miR-200c and miR-141, for example, regulate invasion and metastasis in colorectal cancer via altered miR-200c target gene expression; miR-375 is downregulated in tamoxifen-resistant breast cancer cells with EMT-like properties, and its reexpression partly reverses EMT [51,52]. Other miRNAs that have been discussed to play a central role in EMT are, among others, miR-1, 9, 24, 29b, 30a, 31, 124, 155, 192/215 and 661 (reviewed in [6]). Their mechanisms of action include post-transcriptional regulation of “EMT master genes” or of genes defining the epithelial or mesenchymal phenotype of the cell (such as E-/N-cadherin or vimentin). However, a thorough review of the role of miRNAs in EMT and their clinical significance would lie beyond the scope of this text, where we would like focus on the role of well-characterized proteins in EMT.

### Tissue markers of EMT

Unlike the various mechanisms that are known to initiate or repress EMT, the observed hallmarks of established or ongoing EMT are quite consistent. As previously mentioned, loss or degradation of proteins associated with epithelial homeostasis, cell polarity and cell adhesion, such as E-cadherin, RhoA or Plakophilin 2 is frequently observed in EMT (Figure 1A); some proteins that play key roles in cell-cell adhesion when attached to the membrane, such as β-catenin, are redistributed to the cytoplasm [11,12,17]. Moreover, cells undergoing EMT show decreased expression of epithelial cytokeratin filaments, such as keratins 8 and 18 [53]. On the other hand, the intermediate filament protein Vimentin is frequently overexpressed and contributes to cell migration as well as invasion-associated gene expression by stabilizing the phosphorylated state of MAPK and is thus regarded as a stable marker of EMT; moreover, its presence is a prerequisite for the maturation of invadopodia which are indispensable for cell invasion [32,54,55]. Dysregulated expression of transcription factors, such as Notch1, Slug, Snail, Twist or Zeb1 has been described in invasive tumors displaying EMT; these markers are therefore designated as “EMT master genes”. Table 1 provides an overview over selected dysregulated protein markers that have been and still are frequently used in the assessment of EMT.

**Table 1:** Frequently used protein markers for epithelial-mesenchymal transition (EMT)

**Marker**	**Original function**	**Tissue**	**Reference**
**Downregulated in EMT**			
α-catenin	Cell adhesion molecule	Lung	[56]
β-catenin (membrane)^1^	Cell adhesion molecule	Colon, Pancreas (NET)	[57,58]
Claudin	Cell adhesion molecule	Esophagus, Breast	[59,60]
Cytokeratins	Cytoskeletal filament	Lung, Esophagus	[16,61,62]
E-cadherin	Cell adhesion molecule	Colon, Breast, Lung, Ovary, Esophagus, Prostate, Cervix	[16,61,63-70]
Occludin	Cell adhesion molecule	Ovary	[18,71]
**Upregulated in EMT**			
Brachyury	Transcription factor	Pancreas, Breast, Lung	[72]
β-catenin (cytoplasm/ nucleus)^1^	Transcription factor	Breast, Cervix	[73]
EGFR	Tyrosine kinase receptor	Cervix	[70]
N-cadherin	Cell adhesion molecule	Ovary, Prostate	[68,74]
Notch-1	Transcription factor	Prostate	[75]
p16^INK4a^	Cell cycle regulator	Colon, Urothelium	[23,76]
Slug	Transcription factor	Breast, Ovary	[11,77]
Snail	Transcription factor	Breast, Cervix, Ovary	[11,70,77]
TTF-1	Transcription factor	Lung	[61]
Twist	Transcription factor	Breast, Stomach	[78,79]
Vimentin	Cytoskeletal filament	Breast, Esophagus, Cervix	[16,55,62,70]
ZEB1	Transcription factor	Colon, Breast, Ovary	[51,68,80,81]

^1^Membraneous depletion, but cytoplasmic accumulation/nuclear translocation.

NET, neuroendocrine tumor; EGFR, epidermal growth factor receptor; TTF-1, thyroid transcription factor-1; ZEB1, Zinc finger E-box-binding homeobox 1.

### EMT, tumor invasion and metastasis

The highest clinical significance of the EMT process is linked to its role in tumor cell invasion and metastasis. In a transgenic mouse model of pancreatic beta-cell carcinogenesis, the switch from noninvasive adenoma to invasive carcinoma is associated with a loss of E-cadherin expression [82]; moreover, it has been shown that loss of membraneous β-catenin is associated with tumor cell budding, a morphologic hallmark of invasive tumor phenotype and tumor aggressivity in colorectal cancer tissue specimens [83-85]. In samples from 49 breast cancer patients, the single-cell infiltration pattern that is observed in some lobular carcinomas has been linked to protein truncation mutations in the *CDH1* gene encoding for E-cadherin [86], and hypoxia-induced upregulation of Slug and Snail is associated with increased breast cancer cell migration and invasion *in vitro*[77]. Accordingly, expression of Vimentin can be found in many aggressive breast cancer cell lines [87]. As mentioned above, to allow for tumor cell invasion into the vasculature as a prerequisite for metastatic seeding, EMT cooperates with invadopodia formation and MMP activity [36,37]; circulating tumor cells (CTCs) obtained from peripheral blood of breast cancer patients frequently show an EMT-like phenotype [88,89]. In human and murine malignant melanoma cells, metastatic dissemination is enhanced and accelerated via Snail-induced EMT [90], and bone metastases of human prostate carcinomas show significant overexpression of Notch-1 compared to the primary tumors [75]. In lung carcinoma surgical specimens, tumor dedifferentiation as well as lymphogenous metastasis are also associated with reduced E-cadherin expression [91].

However, as mentioned above, some authors also reported reexpression of epithelial markers, such as E-cadherin, along with loss of EMT-associated transcription factors in established metastases [41]. This apparent reversal of EMT, often referred to as mesenchymal-epithelial transition (MET), has been described for metastases of colorectal carcinoma, non-small cell lung cancer and transitional cell carcinoma [92-94]. There is an ongoing debate regarding the extent to which these findings reflect a basic mechanism in the establishment of metastases or if they are restricted to certain tumor entities or reflect distinct circumjacent conditions [4,41]. There are also critical voices that doubt the role of EMT in invasion at all, since in most histopathologic specimens, many tumors invade and metastasize by cohesive and multicellular rather than single-cell migration, and histopathologists rarely see abundant mesenchymal-like tumor cells in routine surgical specimens [4,95,96]. This apparent contradiction might in part be explained by regarding EMT as a transient state of a small proportion of migrating tumor cells, with only single tumor cells or small clusters of cells obtaining the ideal dynamic configuration for different stages of invasion and metastasis; this reasonable compromise has been referred to as “spatial and temporal heterogeneity of EMT” by Voulgari et al. (Figure 1) [97,98].

Notably, there is another controversy regarding the point whether the EMT program is associated with enhanced or attenuated proliferative activity of the cell. While under normal circumstances TGF-β signaling exerts an anti-proliferative and pro-apoptotic effect, there is experimental evidence that tumor cells having undergone EMT do in fact show enhanced proliferation and resistance to apoptosis [99,100]. This apparent contradiction might also be explained by a possible heterogeneity in the course and the extent of EMT, with specialized cell populations exerting different roles during invasion and metastasis; this is in line with findings that highly metastatic breast cancer cells in fact show strong activity of the TGF-β signaling pathway [101]. It has also been proposed that the two oppositional endpoints of TGF-β signaling might be distinguished by loss of Smad4 in tumor tissue, which promotes TGF-β-mediated tumorigenesis, while in parallel abolishing its tumor-suppressive functions [102]. Additionally, as described above, signaling along various non-TGF-β-dependent pathways might be capable of overcoming the original anti-tumorigenic effect of TGF-β in the course of an “unfriendly takeover” of central TGF-β signaling nodes and target genes; concurrent PI3K/AKT signaling, for example, thwarts the pro-apoptotic effect of TGF-β, thus selectively allowing for the pro-metastatic effects of the pathway to occur [13,49,50,77].

### EMT, cancer stem cells and therapy resistance

Concerning the role of EMT in antitumoral therapies, it has been shown that an EMT-like cellular phenotype in both surgical specimens and cell lines is associated with increased resistance to most conventional approaches, such as chemotherapy [103-105], radiotherapy [106] or hormone withdrawal [107,108]. The observed changes in gene expression during EMT show striking similarity to a rather dedifferentiated state of the cell; in immortalized mammary epithelial cells, induction of EMT not only leads to the gain of a mesenchymal phenotype, but also induces the expression of certain stem cell markers (CD44^+^/CD24^−^) [109]. This generation of breast cancer cells with both cancer stem cell and mesenchymal-like characteristics has again been shown to be dependent on an activation Ras/MAPK signaling, and the link between EMT and cancer cell “stemness” is supported by the fact that genes associated with angiogenesis, invasion and metastasis are overexpressed in stem cell- like CD44^+^/CD24^−^ breast cancer cells; notably, after chemotherapy for breast cancer, residual tumor cells frequently display a stem cell-like phenotype and increased mammosphere formation efficiency [101,110,111]. The sensitivity of non-small cell lung cancer cells to EGFR kinase inhibition depends on their respective EMT phenotype, with mesenchymal-like cells (that express Vimentin or Fibronectin) being less sensitive to EGFR inhibition [112]. In the NSCLC model, it has also been shown that this resistance might be mediated via EGFR-independent MEK-Erk pathway activation and PDGFR, FGFR and TGF-β receptor acquisition in mesenchymal-like tumor cells [113]. An EMT-like gene expression profile in lung cancer cell lines is in fact associated with increased resistance to both EGFR and PI3K/Akt pathway inhibitors, a finding that could even be confirmed in a small patient cohort [114]. Thus, the mechanisms of resistance to antineoplastic therapies might be due to stem-cell like properties of tumor cells that have undergone EMT, allowing for self-renewing of a proportion of cells within the tumor based on the activation of central signaling pathways that are common to both processes, such as TGF-β, wnt, Notch and Hedgehog [13]. Associations between EMT-like properties and a stem-cell like cellular phenotype have not only been described in carcinoma of the breast and in NSCLC, but also in urinary bladder, head and neck, pancreas, and colorectal carcinoma; here, increased resistance to anti-epithelial growth factor receptor (EGFR)-directed therapy is also associated with an EMT-like phenotype of the tumor cells [115].

### EMT and patient prognosis

Since the metastatic spread of malignant tumors accounts for the majority of cancer-specific deaths [116-118], possible correlations between EMT markers and patient prognosis have been intensely studied in multiple tumor entities. However, there is still controversy regarding the impact of the EMT concept on the actual situation in human malignancies [119]. Therefore, much effort has been put into linking the expression of EMT markers to data on patient survival. In colon cancer, the upregulation of genes involved in EMT/matrix remodeling defines a molecularly distinct subtype with very unfavorable prognosis; downregulation of E-cadherin in patient samples, on the other hand, seems to be associated with high TNM stages and distant metastasis [120,121]. Accordingly, basal-like, triple-negative breast cancers that show upregulation of Vimentin have a poor prognosis [54,87]. In a meta-analysis of 1107 breast cancer samples, Tobin et al. showed reduced recurrence free survival in tumors displaying increased gene expression of EMT markers *SNAI2, TWIST1* and *VIM,* and decreased levels of *CDH1* (encoding for E-cadherin) [122]. In contrast, only recently Lee et al. were unable to confirm an impact of the tissue expression of EMT markers on disease-free survival or overall survival in breast cancer patients [123]. In prostate cancer, expression levels of EMT markers Twist and Vimentin - as assessed by immunohistochemistry in radical prostatectomy specimens - are independent predictors for biochemical recurrence as defined by a resurgence in serum prostate-specific antigen (PSA) levels following surgery [124]. Additionally, loss of membraneous E-cadherin staining seems to be associated with increased Gleason score, advanced clinical stage, and poor prognosis in prostate cancer [125]. In tissue samples from 354 primary tumors and 30 metastases of endometrial carcinomas, Tanaka et al. reported that EMT status (E-cadherin-negative/ Snail-positive immunostaining) correlated with histological type, FIGO stage, myometrial invasion and positive peritoneal cytology while it was inversely associated with both progression-free survival (HR = 0.443) and overall survival (HR = 0.366) [126].

Taken together, numerous studies in a variety of tumor entities show statistical correlations between patient prognosis and alterations of various markers compatible with EMT. However, it may be difficult to yield reliable prognostic information for an individual patient from the expression pattern of EMT markers in surgical specimens; this is in part due to high variability of marker expression patterns in different tumor areas in a heterogeneous sample [127,128]. Moreover, artificial induction of EMT *in vitro* (under certain cell culture conditions) as well as *in vivo* (in surgical specimens subjected to ischemia) has been shown [129,130]. Another key problem is the lack of a standardized diagnostic definition of which gene or which extent of expression changes is sufficient to determine EMT; in many reports, expression changes of one or two genes are already referred to as EMT or “partial EMT”, thus impairing the comparability of studies [4,131]. Furthermore, as has already been discussed above, it is still unclear whether the gene expression changes observed in EMT reflect “passenger mutations” caused by genetic instability during tumor dedifferentiation rather than a real mesenchymal transdifferentiation state of the cell [4]. From this point of view, the expression of EMT markers simply represents a more primitive differentiation state of the cancer cell that is associated with oncogenic activation of a variety of signaling molecules [132].

### EMT as a potential target for antineoplastic therapies

Since the population of stem cell-like tumor cells will always bear considerable resistance to conventional therapies and since the hallmarks of EMT have been identified in a significant proportion of these cells as described above, efforts have been made to develop antineoplastic therapies that directly target EMT. The aim of most therapeutic approaches is to block or slow down invasion and metastasis in tumors or, in benign conditions associated with EMT, impede fibrotic organ remodeling [96,133]. Table 2 shows some current therapeutic approaches that are aiming at EMT, most of them targeting kinase signaling pathways upstream EMT master gene expression. In mouse hepatocytes that have undergone EMT, it has been shown that inhibition of STAT3 signaling, for example, reduces EMT-like changes; in renal tubular epithelium, ALK receptor activation via recombinant BMP-7 acts antagonistically to TGF-β and leads to reexpression of E-cadherin [134-136]. Inhibition of kinase signaling downstream FGFR3, ILK, Ras/MAPK or PI3K/AKT downregulates tumor formation, EMT master gene expression and invasive potential in colorectal, lung and pancreatic carcinoma cells *in vitro*, while in some models, re-expression of E-cadherin could be shown upon treatment with kinase inhibitors [137-140]. In a mouse model of hepatocellular carcinoma, transformation with kinase-inactivated integrin-linked kinase (KI-ILK) partially restored the sensitivity to anti-EGFR treatment [141]. Accordingly, our own group showed that Ras-driven EMT is attenuated via Sorafenib-mediated inhibition of Urokinase plasminogen activator (uPA) expression in RT112 urothelial carcinoma cells [23].

**Table 2:** Therapeutic approaches targeting EMT in benign and malignant processes

**Organ/entity**	**Target**	**Approach**	**Mechanism**	**Effect**	**Ref.**
Liver (Hepatocytes)	STAT3	Sorafenib	Inhibition of STAT3 phosphorylation	TGF-β signaling ↓,	[134]
				Apoptosis ↓,	
				Fibrosis ↓	
Kidney (Tubular epithelium)	ALK3/6 receptors Smad5	Recombinant BMP-7	Antagonistic ALK receptor activation/Smad1 signaling	E-cadherin ↑	[111, [136]
Colorectal cancer	FGFR4	siRNA Knockdown	Reduction of Src and MEK1/2-ERK1/2 signaling	Tumor formation ↓,	[137]
		Targeting antibodies		Cell growth ↓	
		PD173074, TKI-25		Angiogenesis ↓	
Hepatocellular carcinoma	ILK	Kinase-inactivated ILK (S343A)	Reduction of Akt signaling	Sensitivity to anti-EGFR therapy ↑	[141]
Lung adenocarcinoma	HAT/HDAC	Sorafenib	HAT expression↑	Changes in histone acetylation and transcriptional repression of EMT-related genes	[138]
			HDAC expression↓, possibly via inhibition of Ras/Raf/MAPK and ErbB signaling		
	Brachyury	Vaccination (Brachyury-specific T cells)	T-cell mediated cytotoxicity	Lysis of Brachyury-positive tumor cells	[3]
	Axl RTK	SGI-7079	Inhibition of Axl phosphorylation	Growth of mesenchymal NSCLC xenograft tumors ↓	[114]
Breast cancer	LYN kinase	Dasatinib	Inhibition of LYN kinase activity	Invasion ↓	[142]
	EMT master gene expression	Metformin	Transcriptional repression	Twist1 ↓, ZEB1 ↓,	[143]
				Slug ↓,	
				TGF-β 1–3 ↓,	
				MMP-3, MMP-9 ↓,	
				E-cadherin ↑	
Urothelial carcinoma *in situ* (UCIS)	Urokinase plasminogen activator (uPA) expression	Sorafenib	Inhibition of Ras/MAPK signaling	uPA ↓,	[23]
				E-cadherin ↑	
Pancreatic cancer	Gli1, Ptch (Hedgehog target genes)	Cyclopamine, IPI-269609	Inhibition of Hedgehog signaling	Snail ↓,	[139,140]
				E-cadherin ↑,	
				Metastasis ↓	
	EMT master gene expression	Resveratrol	Transcriptional repression	Slug ↓,	[144]
				Snail ↓,	
				ZEB1↓,	
				Migration/Invasion ↓	
	Axl RTK	siRNA Knockdown	Inhibition of MAPK and PI3K/AKT kinase signaling	GTP-bound Rho/Rac↓,	[145]
				Slug ↓,	
				Snail ↓,	
				Twist ↓,	
				MMP-9 ↓,	
				Migration/Invasion ↓	

STAT3, Signal transducer and activator of transcription 3; TGF-β, transforming growth factor β; ALK3, activin-like kinase 3; BMP-7, bone morphogenetic protein 7, FGFR4, fibroblast growth factor receptor 4; Src, sarcoma kinase; MEK, mitogen-associated protein kinase kinase; ERK, extracellular signal-related kinase; siRNA, small interfering RNA; ILK, integrin-linked kinase; AKT, protein kinase B; HAT, histone acetyltransferase; HDAC, histone deacetylase; EGFR, epidermal growth factor receptor; RTK, receptor tyrosine kinase; NSCLC, non-small cell lung cancer; LYN, Lck/Yes-related novel protein tyrosine kinase; MMP, matrix metalloproteinase; uPA, urokinase plasminogen activator; ZEB1, Zinc finger E-box-binding homeobox 1; PI3K, phosphatidylinositol-3-kinase.

From the knowledge of the diverse kinase-dependent signaling pathways that are activated during EMT, it is not surprising that the application of multi-kinase inhibitors such as Sorafenib is capable of reversing the process to a certain extent. Up to now, concepts that are directly targeting EMT master genes or their effectors are rare. Interesting new approaches include the previously mentioned vaccination against Brachyury-positive tumor cells and the transcriptional repression of EMT master gene expression by the anti-diabetic drug Metformin (Table 2) [3,72,143]. Resveratol, a dietary polyphenol, downregulated expression of EMT master genes Zeb1, Snail and Slug and impaired CSC self-renewal capacity, tumor growth and invasion in a mouse model of pancreatic ductal adenocarcinoma [144]. However, despite the abundance of literature on effectors of EMT, there is a lack of studies that show a solid effect of a specific compound in an *in vivo* system additionally to cell culture data, and to our best knowledge, a study that rescued the EMT phenotype after application of a certain compound - for example by overexpressing an EMT-inducing transcription factor - has so far not been conducted. Therefore, most of the data on drugs targeting EMT has to be regarded as preliminary, and further research is needed to identify valuable pharmacologic targets during the induction or progression of the EMT process.

## Conclusions

Taken together, the concept of EMT is a valuable model for the morphologic and molecular changes observed in tumor cell invasion as well as tissue fibrosis. However, it is still unclear whether or to which extent cells in fact do undergo a complete conversion of cell type or show only transient changes in cellular morphology and protein expression patterns that are supportive of a migratory phenotype. Despite the controversies dealing with the definition and extent of EMT, the association between an EMT-like cellular phenotype - as shown by changes in marker protein expression - and tumor aggressivity has been well-proven in a variety of malignancies. In recent years, first promising results have been reported concerning a possible use of the EMT process as a pharmacological target, especially with multi-kinase inhibitors such as Sorafenib. However, since most of these results are actually derived from *in vitro* data and definite proof of druggable EMT *in vivo* is still missing, the clinical utility of these approaches remains to be elucidated in future studies.

## Abbreviations

AJ: Adherens junction; CXCR-1: CXC motif chemokine receptor 1/Interleukin-8-receptor alpha; ECM: Extracellular matrix; EMT: Epithelial-mesenchymal transition; FGFR: Fibroblast growth factor receptor; FIGO: Fédération internationale de Gynécologie et d’Obstétrique; GSK3β: Glycogen synthase kinase 3β; HDAC: Histone deacetylase; HIF: Hypoxia-inducible factor; MAPK: Mitogen-associated protein kinase; MEK: Mitogen-associated protein kinase kinase; MET: Mesenchymal-epithelial transition; MMP: Matrix metalloproteinase; NSCLC: Non-small cell lung cancer; PAR: Partitioning defective; PDGFR: Platelet-derived growth factor receptor; PI3K: Phosphatidylinositol-3-kinase; RTK: Receptor tyrosine kinase; TGF-β: Transforming growth factor β; TNM: Tumor/Nodes/Metastasis (clinical classification system for tumor spread); uPA: Urokinase plasminogen activator; ZO-1: Zonula occludens 1.

## Competing interests

Prof AJ Schrader receives compensation as a consultant for Bayer Healthcare AG, which manufactures Sorafenib (Nexavar®) for clinical application.

## Authors’ contributions

All authors participated in the design of this review. KS, SE and JS reviewed literature on the molecular basis of EMT, on EMT markers and on EMT in tumor invasion and metastasis; KS and AJS reviewed literature on the association between EMT and cancer stem cells, therapy resistance and patient prognosis as well as on EMT as a pharmaceutical target. All authors read and approved the final manuscript.

## References

[B1] Greenburg G Hay ED Epithelia suspended in collagen gels can lose polarity and express characteristics of migrating mesenchymal cells J Cell Biol 1982 3 333 339 7142291 10.1083/jcb.95.1.333 PMC2112361

[B2] Guarino M Rubino B Ballabio G The role of epithelial-mesenchymal transition in cancer pathology Pathology 2007 3 305 318 17558857 10.1080/00313020701329914

[B3] Palena C Fernando RI Hamilton DH An immunotherapeutic intervention against tumor progression: targeting a driver of the epithelial-to-mesenchymal transition Oncoimmunology 2014 3 e27220 24575384 10.4161/onci.27220 PMC3929358

[B4] Chui MH Insights into cancer metastasis from a clinicopathologic perspective: epithelial-mesenchymal transition is not a necessary step Int J Cancer 2013 3 1487 1495 22833228 10.1002/ijc.27745

[B5] Kalluri R Weinberg RA The basics of epithelial-mesenchymal transition J Clin Invest 2009 3 1420 19487818 10.1172/JCI39104 PMC2689101

[B6] Lamouille S Xu J Derynck R Molecular mechanisms of epithelial–mesenchymal transition Nat Rev Mol Cell Biol 2014 3 178 196 24556840 10.1038/nrm3758 PMC4240281

[B7] Zhang H Liu L Wang Y Zhao G Xie R Liu C Xiao X Wu K Nie Y Zhang H Fan D KLF8 involves in TGF-beta-induced EMT and promotes invasion and migration in gastric cancer cells J Cancer Res Clin Oncol 2013 3 1033 1042 23504025 10.1007/s00432-012-1363-3 PMC11824695

[B8] Vittal R Fan L Greenspan DS Mickler EA Gopalakrishnan B Gu H Benson HL Zhang C Burlingham W Cummings OW IL-17 induces type V collagen overexpression and EMT via TGF-β-dependent pathways in obliterative bronchiolitis Am J Physiol Lung Cell Mol Physiol 2013 3 L401 L414 23262228 10.1152/ajplung.00080.2012 PMC3602743

[B9] Massagué J TGFβ in cancer Cell 2008 3 215 23018662538

[B10] Shi Y Massagué J Mechanisms of TGF-β signaling from cell membrane to the nucleus Cell 2003 3 685 700 12809600 10.1016/s0092-8674(03)00432-x

[B11] Elloul S Bukholt Elstrand M Nesland JM Tropé CG Kvalheim G Goldberg I Reich R Davidson B Snail, slug, and smad-interacting protein 1 as novel parameters of disease aggressiveness in metastatic ovarian and breast carcinoma Cancer 2005 3 1631 1643 15742334 10.1002/cncr.20946

[B12] Thiery JP Huang R Linking epithelial-mesenchymal transition to the well-known polarity protein Par6 Dev Cell 2005 3 456 458 15809027 10.1016/j.devcel.2005.03.002

[B13] Singh A Settleman J EMT, cancer stem cells and drug resistance: an emerging axis of evil in the war on cancer Oncogene 2010 3 4741 4751 20531305 10.1038/onc.2010.215 PMC3176718

[B14] Hazan RB Qiao R KEREN R BADANO I SUYAMA K Cadherin switch in tumor progression Ann N Y Acad Sci 2004 3 155 163 15153430 10.1196/annals.1294.016

[B15] Toivola DM Tao G-Z Habtezion A Liao J Omary MB Cellular integrity plus: organelle-related and protein-targeting functions of intermediate filaments Trends Cell Biol 2005 3 608 617 16202602 10.1016/j.tcb.2005.09.004

[B16] Lorenz KJ Kraft K Graf F Pröpper C Steinestel K The role of reflux-induced epithelial-mesenchymal transition in periprosthetic leakage after prosthetic voice rehabilitation Head Neck 2014 Advance online publication 9 April 2014 10.1002/hed.23622 24532155

[B17] Huang RY-J Guilford P Thiery JP Early events in cell adhesion and polarity during epithelial-mesenchymal transition J Cell Sci 2012 3 4417 4422 23165231 10.1242/jcs.099697

[B18] Ikenouchi J Matsuda M Furuse M Tsukita S Regulation of tight junctions during the epithelium-mesenchyme transition: direct repression of the gene expression of claudins/occludin by Snail J Cell Sci 2003 3 1959 1967 12668723 10.1242/jcs.00389

[B19] Ohkubo T Ozawa M The transcription factor snail downregulates the tight junction components independently of E-cadherin downregulation J Cell Sci 2004 3 1675 1685 15075229 10.1242/jcs.01004

[B20] De Craene B Berx G Regulatory networks defining EMT during cancer initiation and progression Nat Rev Cancer 2013 3 97 110 23344542 10.1038/nrc3447

[B21] Pickup M Novitskiy S Moses HL The roles of TGF [beta] in the tumour microenvironment Nat Rev Cancer 2013 3 788 799 24132110 10.1038/nrc3603 PMC4025940

[B22] Mulholland DJ Kobayashi N Ruscetti M Zhi A Tran LM Huang J Gleave M Wu H Pten loss and RAS/MAPK activation cooperate to promote EMT and metastasis initiated from prostate cancer stem/progenitor cells Cancer Res 2012 3 1878 1889 22350410 10.1158/0008-5472.CAN-11-3132 PMC3319847

[B23] Steinestel J Cronauer MV Müller J Al Ghazal A Skowronek P Arndt A Kraft K Schrader M Schrader AJ Steinestel K Overexpression of p16INK4a in urothelial carcinoma in situ is a marker for MAPK-mediated epithelial-mesenchymal transition but is not related to human papillomavirus infection PLoS One 2013 3 e65189 23724131 10.1371/journal.pone.0065189 PMC3665800

[B24] Bourboulia D Stetler-Stevenson WG Matrix metalloproteinases (MMPs) and tissue inhibitors of metalloproteinases (TIMPs): positive and negative regulators in tumor cell adhesion Seminars in cancer biology 2010 Amsterdam: Elsevier 161 168 10.1016/j.semcancer.2010.05.002 PMC2941566 20470890

[B25] Gerhardt H Golding M Fruttiger M Ruhrberg C Lundkvist A Abramsson A Jeltsch M Mitchell C Alitalo K Shima D VEGF guides angiogenic sprouting utilizing endothelial tip cell filopodia J Cell Biol 2003 3 1163 1177 12810700 10.1083/jcb.200302047 PMC2172999

[B26] Ridley AJ Life at the leading edge Cell 2011 3 1012 1022 21703446 10.1016/j.cell.2011.06.010

[B27] Chen Y-S Huang W-L Chang S-H Chang K-W Kao S-Y Lo J-F Su P-F Enhanced filopodium formation and stem-like phenotypes in a novel metastatic head and neck cancer cell model Oncol Rep 2013 3 2829 2837 24100418 10.3892/or.2013.2772

[B28] Gulhati P Bowen KA Liu J Stevens PD Rychahou PG Chen M Lee EY Weiss HL O’Connor KL Gao T mTORC1 and mTORC2 regulate EMT, motility, and metastasis of colorectal cancer via RhoA and Rac1 signaling pathways Cancer Res 2011 3 3246 3256 21430067 10.1158/0008-5472.CAN-10-4058 PMC3085654

[B29] Eckert MA Lwin TM Chang AT Kim J Danis E Ohno-Machado L Yang J Twist1-induced invadopodia formation promotes tumor metastasis Cancer Cell 2011 3 372 386 21397860 10.1016/j.ccr.2011.01.036 PMC3072410

[B30] Steinestel K Brüderlein S Lennerz JK Steinestel J Kraft K Pröpper C Meineke V Möller P Expression and Y435-phosphorylation of Abelson interactor 1 (Abi1) promotes tumour cell adhesion, extracellular matrix degradation and invasion by colorectal carcinoma cells Mol Cancer 2014 3 145 24913355 10.1186/1476-4598-13-145 PMC4066275

[B31] Kardassis D Murphy C Fotsis T Moustakas A Stournaras C Control of transforming growth factor β signal transduction by small GTPases FEBS J 2009 3 2947 2965 19490100 10.1111/j.1742-4658.2009.07031.x

[B32] Schoumacher M Goldman RD Louvard D Vignjevic DM Actin, microtubules, and vimentin intermediate filaments cooperate for elongation of invadopodia J Cell Biol 2010 3 541 556 20421424 10.1083/jcb.200909113 PMC2867303

[B33] Deryugina E Behrendt N Experimental approaches for understanding the role of matrix metalloproteinases in cancer invasion Matrix Proteases in Health and Disease 2012 Weinheim: Wiley-VCH Verlag 181 211

[B34] Lin CY Tsai PH Kandaswami CC Lee PP Huang CJ Hwang JJ Lee MT Matrix metalloproteinase-9 cooperates with transcription factor Snail to induce epithelial–mesenchymal transition Cancer Sci 2011 3 815 827 21219539 10.1111/j.1349-7006.2011.01861.x

[B35] Shah PP Fong MY Kakar SS PTTG induces EMT through integrin αVβ3-focal adhesion kinase signaling in lung cancer cells Oncogene 2011 3 3124 3135 22081074 10.1038/onc.2011.488 PMC3288952

[B36] Yoo YA Kang MH Lee HJ B-h K Park JK Kim HK Kim JS Oh SC Sonic hedgehog pathway promotes metastasis and lymphangiogenesis via activation of Akt, EMT, and MMP-9 pathway in gastric cancer Cancer Res 2011 3 7061 7070 21975935 10.1158/0008-5472.CAN-11-1338

[B37] Karihtala P Auvinen P Kauppila S Haapasaari K-M Jukkola-Vuorinen A Soini Y Vimentin, zeb1 and Sip1 are up-regulated in triple-negative and basal-like breast cancers: association with an aggressive tumour phenotype Breast Cancer Res Treat 2013 3 81 90 23412770 10.1007/s10549-013-2442-0

[B38] Nisticò P Bissell MJ Radisky DC Epithelial-mesenchymal transition: general principles and pathological relevance with special emphasis on the role of matrix metalloproteinases Cold Spring Harb Perspect Biol 2012 3 a011908 22300978 10.1101/cshperspect.a011908 PMC3281569

[B39] Radisky DC Levy DD Littlepage LE Liu H Nelson CM Fata JE Leake D Godden EL Albertson DG Nieto MA Rac1b and reactive oxygen species mediate MMP-3-induced EMT and genomic instability Nature 2005 3 123 127 16001073 10.1038/nature03688 PMC2784913

[B40] Ocaña Oscar H Córcoles R Fabra Á Moreno-Bueno G Acloque H Vega S Barrallo-Gimeno A Cano A Nieto MA Metastatic colonization requires the repression of the epithelial-mesenchymal transition inducer Prrx1 Cancer Cell 2012 3 709 724 23201163 10.1016/j.ccr.2012.10.012

[B41] Tsai Jeff H Donaher Joana L Murphy Danielle A Chau S Yang J Spatiotemporal regulation of epithelial-mesenchymal transition is essential for squamous cell carcinoma metastasis Cancer Cell 2012 3 725 736 23201165 10.1016/j.ccr.2012.09.022 PMC3522773

[B42] Higgins DF Kimura K Bernhardt WM Shrimanker N Akai Y Hohenstein B Saito Y Johnson RS Kretzler M Cohen CD Eckardt KU Iwano M Haase VH Hypoxia promotes fibrogenesis in vivo via HIF-1 stimulation of epithelial-to-mesenchymal transition J Clin Invest 2007 3 3810 3820 18037992 10.1172/JCI30487 PMC2082142

[B43] Sahlgren C Gustafsson MV Jin S Poellinger L Lendahl U Notch signaling mediates hypoxia-induced tumor cell migration and invasion Proc Natl Acad Sci U S A 2008 3 6392 6397 18427106 10.1073/pnas.0802047105 PMC2359811

[B44] Kaidi A Williams AC Paraskeva C Interaction between beta-catenin and HIF-1 promotes cellular adaptation to hypoxia Nat Cell Biol 2007 3 210 217 17220880 10.1038/ncb1534

[B45] Yang F Sun L Li Q Han X Lei L Zhang H Shang Y SET8 promotes epithelial–mesenchymal transition and confers TWIST dual transcriptional activities EMBO J 2012 3 110 123 21983900 10.1038/emboj.2011.364 PMC3252577

[B46] Luo D Wang J Li J Post M Mouse snail is a target gene for HIF Mol Cancer Res 2011 3 234 245 21257819 10.1158/1541-7786.MCR-10-0214

[B47] Imai T Horiuchi A Wang C Oka K Ohira S Nikaido T Konishi I Hypoxia attenuates the expression of E-cadherin via up-regulation of SNAIL in ovarian carcinoma cells Am J Pathol 2003 3 1437 1447 14507651 10.1016/S0002-9440(10)63501-8 PMC1868286

[B48] Bates RC DeLeo Iii MJ Mercurio AM The epithelial–mesenchymal transition of colon carcinoma involves expression of IL-8 and CXCR-1-mediated chemotaxis Exp Cell Res 2004 3 315 324 15350531 10.1016/j.yexcr.2004.05.033

[B49] Wu Y Ginther C Kim J Mosher N Chung S Slamon D Vadgama JV Expression of Wnt3 activates Wnt/β-catenin pathway and promotes EMT-like phenotype in trastuzumab-resistant HER2-overexpressing breast cancer cells Mol Cancer Res 2012 3 1597 1606 23071104 10.1158/1541-7786.MCR-12-0155-T PMC3732195

[B50] Vincan E Barker N The upstream components of the Wnt signalling pathway in the dynamic EMT and MET associated with colorectal cancer progression Clin Exp Metastasis 2008 3 657 663 18350253 10.1007/s10585-008-9156-4

[B51] Hur K Toiyama Y Takahashi M Balaguer F Nagasaka T Koike J Hemmi H Koi M Boland CR Goel A MicroRNA-200c modulates epithelial-to-mesenchymal transition (EMT) in human colorectal cancer metastasis Gut 2013 3 1315 1326 22735571 10.1136/gutjnl-2011-301846 PMC3787864

[B52] Ward A Balwierz A Zhang JD Kublbeck M Pawitan Y Hielscher T Wiemann S Sahin O Re-expression of microRNA-375 reverses both tamoxifen resistance and accompanying EMT-like properties in breast cancer Oncogene 2013 3 1173 1182 22508479 10.1038/onc.2012.128

[B53] Fortier A-M Asselin E Cadrin M Keratin 8 and 18 loss in epithelial cancer cells increases collective cell migration and cisplatin sensitivity through Claudin1 Up-regulation J Biol Chem 2013 3 11555 11571 23449973 10.1074/jbc.M112.428920 PMC3630871

[B54] Vuoriluoto K Haugen H Kiviluoto S Mpindi JP Nevo J Gjerdrum C Tiron C Lorens JB Ivaska J Vimentin regulates EMT induction by Slug and oncogenic H-Ras and migration by governing Axl expression in breast cancer Oncogene 2011 3 1436 1448 21057535 10.1038/onc.2010.509

[B55] Sohal SS Soltani Abhari A Weston S Wood-Baker R Walters E de Mello RA Intermediate filament vimentin and potential role in epithelial mesenchymal transition (EMT) Vimentin Concepts and Molecular Mechanisms 2013 New York: Nova Publishers 37 61

[B56] Hirano S Kimoto N Shimoyama Y Hirohashi S Takeichi M Identification of a neural alpha-catenin as a key regulator of cadherin function and multicellular organization Cell 1992 3 293 301 1638632 10.1016/0092-8674(92)90103-j

[B57] Williams CS Zhang B Smith JJ Jayagopal A Barrett CW Pino C Russ P Presley SH Peng D Rosenblatt DO BVES regulates EMT in human corneal and colon cancer cells and is silenced via promoter methylation in human colorectal carcinoma J Clin Invest 2011 3 4056 21911938 10.1172/JCI44228 PMC3195453

[B58] Galván JA Astudillo A Vallina A Fonseca PJ Gómez-Izquierdo L García-Carbonero R González MV Epithelial-mesenchymal transition markers in the differential diagnosis of gastroenteropancreatic neuroendocrine tumors Am J Clin Pathol 2013 3 61 72 23765535 10.1309/AJCPIV40ISTBXRAX

[B59] Lioni M Brafford P Andl C Rustgi A El-Deiry W Herlyn M Smalley KS Dysregulation of claudin-7 leads to loss of E-cadherin expression and the increased invasion of esophageal squamous cell carcinoma cells Am J Pathol 2007 3 709 721 17255337 10.2353/ajpath.2007.060343 PMC1851859

[B60] Kominsky SL Argani P Korz D Evron E Raman V Garrett E Rein A Sauter G Kallioniemi OP Sukumar S Loss of the tight junction protein claudin-7 correlates with histological grade in both ductal carcinoma in situ and invasive ductal carcinoma of the breast Oncogene 2003 3 2021 2033 12673207 10.1038/sj.onc.1206199

[B61] Shi Y Wu H Zhang M Ding L Meng F Fan X Expression of the epithelial-mesenchymal transition-related proteins and their clinical significance in lung adenocarcinoma Diagn Pathol 2013 3 89 23706092 10.1186/1746-1596-8-89 PMC3671218

[B62] Kagalwalla AF Akhtar N Woodruff SA Rea BA Masterson JC Mukkada V Parashette KR Du J Fillon S Protheroe CA Eosinophilic esophagitis: epithelial mesenchymal transition contributes to esophageal remodeling and reverses with treatment J Allergy Clin Immunol 2012 3 1387 1396 e1387 22465212 10.1016/j.jaci.2012.03.005 PMC3340537

[B63] van Roy F Beyond E-cadherin: roles of other cadherin superfamily members in cancer Nat Rev Cancer 2014 3 121 134 24442140 10.1038/nrc3647

[B64] Pichler M Ress AL Winter E Stiegelbauer V Karbiener M Schwarzenbacher D Scheideler M Ivan C Jahn SW Kiesslich T Gerger A Bauernhofer T Calin GA Hoefler G MiR-200a regulates epithelial to mesenchymal transition-related gene expression and determines prognosis in colorectal cancer patients Br J Cancer 2014 3 1614 1621 24504363 10.1038/bjc.2014.51 PMC3960623

[B65] Zheng H Li W Wang Y Xie T Cai Y Wang Z Jiang B miR-23a inhibits E-cadherin expression and is regulated by AP-1 and NFAT4 complex during Fas-induced EMT in gastrointestinal cancer Carcinogenesis 2014 3 173 183 23929433 10.1093/carcin/bgt274

[B66] Shah P Gau Y Sabnis G Histone deacetylase inhibitor entinostat reverses epithelial to mesenchymal transition of breast cancer cells by reversing the repression of E-cadherin Breast Cancer Res Treat 2014 3 99 111 24305977 10.1007/s10549-013-2784-7

[B67] Jin L Chen J Li L Li C Chen C Li S CRH suppressed TGFβ1-induced epithelial-mesenchymal transition via induction of E-cadherin in breast cancer cells Cell Signal 2014 3 757 765 24412750 10.1016/j.cellsig.2013.12.017

[B68] Huang RYJ Wong MK Tan TZ Kuay KT Ng AHC Chung VY Chu YS Matsumura N Lai HC Lee YF Sim WJ Chai C Pietschmann E Mori S Low JJH Choolani M Thiery P An EMT spectrum defines an anoikis-resistant and spheroidogenic intermediate mesenchymal state that is sensitive to e-cadherin restoration by a src-kinase inhibitor, saracatinib (AZD0530) Cell Death Dis 2013 3 e915 24201814 10.1038/cddis.2013.442 PMC3847320

[B69] Sun Y Wang B-E Leong KG Yue P Li L Jhunjhunwala S Chen D Seo K Modrusan Z Gao W-Q Settleman J Johnson L Androgen deprivation causes epithelial–mesenchymal transition in the prostate: implications for androgen-deprivation therapy Cancer Res 2012 3 527 536 22108827 10.1158/0008-5472.CAN-11-3004

[B70] Lee M-Y Chou C-Y Tang M-J Shen M-R Epithelial-mesenchymal transition in cervical cancer: correlation with tumor progression, epidermal growth factor receptor overexpression, and snail up-regulation Clin Cancer Res 2008 3 4743 4750 18676743 10.1158/1078-0432.CCR-08-0234

[B71] Zhu Y Nilsson M Sundfeldt K Phenotypic plasticity of the ovarian surface epithelium: TGF-β1 induction of epithelial to mesenchymal transition (EMT) in vitro Endocrinology 2010 3 5497 5505 20844000 10.1210/en.2010-0486

[B72] Fernando RI Litzinger M Trono P Hamilton DH Schlom J Palena C The T-box transcription factor Brachyury promotes epithelial-mesenchymal transition in human tumor cells J Clin Invest 2010 3 533 20071775 10.1172/JCI38379 PMC2810072

[B73] Li J Zhou BP Activation of β-catenin and Akt pathways by Twist are critical for the maintenance of EMT associated cancer stem cell-like characters BMC Cancer 2011 3 49 21284870 10.1186/1471-2407-11-49 PMC3040162

[B74] Tanaka H Kono E Tran CP Miyazaki H Yamashiro J Shimomura T Fazli L Wada R Huang J Vessella RL An J Horvath S Gleave M Rettig MB Wainberg ZA Reiter RE Monoclonal antibody targeting of N-cadherin inhibits prostate cancer growth, metastasis and castration resistance Nat Med 2010 3 1414 1420 21057494 10.1038/nm.2236 PMC3088104

[B75] Sethi S Macoska J Chen W Sarkar FH Molecular signature of epithelial-mesenchymal transition (EMT) in human prostate cancer bone metastasis Am J Transl Res 2011 3 90 21139809 PMC2981429

[B76] Dawson H Koelzer VH Karamitopoulou E Economou M Hammer C Muller D-E Lugli A Zlobec I The apoptotic and proliferation rate of tumour budding cells in colorectal cancer outlines a heterogeneous population of cells with various impacts on clinical outcome Histopathology 2014 3 577 584 24111856 10.1111/his.12294

[B77] Chen J Imanaka N Griffin JD Hypoxia potentiates Notch signaling in breast cancer leading to decreased E-cadherin expression and increased cell migration and invasion Br J Cancer 2009 3 351 360 20010940 10.1038/sj.bjc.6605486 PMC2816657

[B78] Lo H-W Hsu S-C Xia W Cao X Shih J-Y Wei Y Abbruzzese JL Hortobagyi GN Hung M-C Epidermal growth factor receptor cooperates with signal transducer and activator of transcription 3 to induce epithelial-mesenchymal transition in cancer cells via up-regulation of TWIST gene expression Cancer Res 2007 3 9066 9076 17909010 10.1158/0008-5472.CAN-07-0575 PMC2570961

[B79] Yang Z Zhang X Gang H Li X Li Z Wang T Han J Luo T Wen F Wu X Up-regulation of gastric cancer cell invasion by Twist is accompanied by N-cadherin and fibronectin expression Biochem Biophys Res Commun 2007 3 925 930 17512904 10.1016/j.bbrc.2007.05.023

[B80] Sánchez-Tilló E de Barrios O Siles L Amendola PG Darling DS Cuatrecasas M Castells A Postigo A ZEB1 promotes invasiveness of colorectal carcinoma cells through the opposing regulation of uPA and PAI-1 Clin Cancer Res 2013 3 1071 1082 23340304 10.1158/1078-0432.CCR-12-2675

[B81] Lee J Park M Park J Lee H Shin D Kang Y Lee C Kong G Loss of the polycomb protein Mel-18 enhances the epithelial–mesenchymal transition by ZEB1 and ZEB2 expression through the downregulation of miR-205 in breast cancer Oncogene 2013 3 1325 1335 23474752 10.1038/onc.2013.53

[B82] Perl A-K Wilgenbus P Dahl U Semb H Christofori G A causal role for E-cadherin in the transition from adenoma to carcinoma Nature 1998 3 190 193 9515965 10.1038/32433

[B83] Kevans D Wang LM Sheahan K Hyland J O’Donoghue D Mulcahy H O’Sullivan J Epithelial-mesenchymal transition (EMT) protein expression in a cohort of stage II colorectal cancer patients with characterized tumor budding and mismatch repair protein status Int J Surg Pathol 2011 3 751 760 21791486 10.1177/1066896911414566

[B84] Horcic M Koelzer VH Karamitopoulou E Terracciano L Puppa G Zlobec I Lugli A Tumor budding score based on 10 high-power fields is a promising basis for a standardized prognostic scoring system in stage II colorectal cancer Hum Pathol 2013 3 697 705 23159156 10.1016/j.humpath.2012.07.026

[B85] Ueno H Murphy J Jass J Mochizuki H Talbot I Tumour ‘budding’ as an index to estimate the potential of aggressiveness in rectal cancer Histopathology 2002 3 127 132 11952856 10.1046/j.1365-2559.2002.01324.x

[B86] Berx G Cleton-Jansen A Nollet F De Leeuw W Van de Vijver M Cornelisse C Van Roy F E-cadherin is a tumour/invasion suppressor gene mutated in human lobular breast cancers EMBO J 1995 3 6107 8557030 10.1002/j.1460-2075.1995.tb00301.x PMC394735

[B87] Neve RM Chin K Fridlyand J Yeh J Baehner FL Fevr T Clark L Bayani N Coppe J-P Tong F Speed T Spellman PT DeVries S Lapuk A Wang NJ Kuo WL Stilwell JL Pinkel D Albertson DG Waldman FM McCormick F Dickson RB Johnson MD Lippman M Ethier S Gazdar A Gray JW A collection of breast cancer cell lines for the study of functionally distinct cancer subtypes Cancer Cell 2006 3 515 527 17157791 10.1016/j.ccr.2006.10.008 PMC2730521

[B88] Burgess DJ Breast cancer: circulating and dynamic EMT Nat Rev Cancer 2013 3 148 149 23407577 10.1038/nrc3475

[B89] Yu M Bardia A Wittner BS Stott SL Smas ME Ting DT Isakoff SJ Ciciliano JC Wells MN Shah AM Circulating breast tumor cells exhibit dynamic changes in epithelial and mesenchymal composition Science 2013 3 580 584 23372014 10.1126/science.1228522 PMC3760262

[B90] Kudo-Saito C Shirako H Takeuchi T Kawakami Y Cancer Metastasis Is Accelerated through Immunosuppression during Snail-Induced EMT of Cancer Cells Cancer Cell 2009 3 195 206 19249678 10.1016/j.ccr.2009.01.023

[B91] Sulzer MA Leers MPG van Noord JA Bollen ECM Theunissen PHMH Reduced E-cadherin expression is associated with increased lymph node metastasis and unfavorable prognosis in non-small cell lung cancer Am J Respir Crit Care Med 1998 3 1319 1323 9563756 10.1164/ajrccm.157.4.9703099

[B92] Brabletz T Hlubek F Spaderna S Schmalhofer O Hiendlmeyer E Jung A Kirchner T Invasion and metastasis in colorectal cancer: epithelial-mesenchymal transition, mesenchymal-epithelial transition, stem cells and β-catenin Cells Tissues Organs 2005 3 56 65 15942193 10.1159/000084509

[B93] Soltermann A Tischler V Arbogast S Braun J Probst-Hensch N Weder W Moch H Kristiansen G Prognostic significance of epithelial-mesenchymal and mesenchymal-epithelial transition protein expression in non–small cell lung cancer Clin Cancer Res 2008 3 7430 7437 19010860 10.1158/1078-0432.CCR-08-0935

[B94] Chaffer CL Brennan JP Slavin JL Blick T Thompson EW Williams ED Mesenchymal-to-epithelial transition facilitates bladder cancer metastasis: role of fibroblast growth factor receptor-2 Cancer Res 2006 3 11271 11278 17145872 10.1158/0008-5472.CAN-06-2044

[B95] Ruiter DJ van Krieken JH van Muijen GN de Waal RM Tumour metastasis: is tissue an issue? Lancet Oncol 2001 3 109 112 11905791 10.1016/S1470-2045(00)00229-1

[B96] Garber K Epithelial-to-mesenchymal transition is important to metastasis, but questions remain J Natl Cancer Inst 2008 3 232 239 18270330 10.1093/jnci/djn032

[B97] Nieto MA Cano A The epithelial–mesenchymal transition under control: global programs to regulate epithelial plasticity Semin Cancer Biol 2012 3 361 368 22613485 10.1016/j.semcancer.2012.05.003

[B98] Voulgari A Pintzas A Epithelial-mesenchymal transition in cancer metastasis: mechanisms, markers and strategies to overcome drug resistance in the clinic Biochim Biophys Acta 2009 3 75 90 19306912 10.1016/j.bbcan.2009.03.002

[B99] Bierie B Moses HL Transforming growth factor beta (TGF-β) and inflammation in cancer Cytokine Growth Factor Rev 2010 3 49 59 20018551 10.1016/j.cytogfr.2009.11.008 PMC2834863

[B100] Gore AJ Deitz SL Palam LR Craven KE Korc M Pancreatic cancer–associated retinoblastoma 1 dysfunction enables TGF-β to promote proliferation J Clin Invest 2014 3 338 352 24334458 10.1172/JCI71526 PMC3871249

[B101] Shipitsin M Campbell LL Argani P Weremowicz S Bloushtain-Qimron N Yao J Nikolskaya T Serebryiskaya T Beroukhim R Hu M Molecular definition of breast tumor heterogeneity Cancer Cell 2007 3 259 273 17349583 10.1016/j.ccr.2007.01.013

[B102] Levy L Hill CS Smad4 dependency defines Two classes of transforming growth factor β (TGF-β) target genes and distinguishes TGF-β-induced epithelial-mesenchymal transition from its antiproliferative and migratory responses Mol Cell Biol 2005 3 8108 8125 16135802 10.1128/MCB.25.18.8108-8125.2005 PMC1234333

[B103] Hollier BG Evans K Mani SA The epithelial-to-mesenchymal transition and cancer stem cells: a coalition against cancer therapies J Mammary Gland Biol Neoplasia 2009 3 29 43 19242781 10.1007/s10911-009-9110-3

[B104] Kajiyama H Shibata K Terauchi M Yamashita M Ino K Nawa A Kikkawa F Chemoresistance to paclitaxel induces epithelial-mesenchymal transition and enhances metastatic potential for epithelial ovarian carcinoma cells Int J Oncol 2007 3 277 284 17611683

[B105] Arumugam T Ramachandran V Fournier KF Wang H Marquis L Abbruzzese JL Gallick GE Logsdon CD McConkey DJ Choi W Epithelial to mesenchymal transition contributes to drug resistance in pancreatic cancer Cancer Res 2009 3 5820 5828 19584296 10.1158/0008-5472.CAN-08-2819 PMC4378690

[B106] Kurrey NK Jalgaonkar SP Joglekar AV Ghanate AD Chaskar PD Doiphode RY Bapat SA Snail and slug mediate radioresistance and chemoresistance by antagonizing p53-mediated apoptosis and acquiring a stem-like phenotype in ovarian cancer cells Stem Cells 2009 3 2059 2068 19544473 10.1002/stem.154

[B107] Kim MR Choi HK Cho KB Kim HS Kang KW Involvement of Pin1 induction in epithelial–mesenchymal transition of tamoxifen-resistant breast cancer cells Cancer Sci 2009 3 1834 1841 19681904 10.1111/j.1349-7006.2009.01260.x PMC11159919

[B108] Singh S Sadacharan S Su S Belldegrun A Persad S Singh G Overexpression of vimentin: role in the invasive phenotype in an androgen-independent model of prostate cancer Cancer Res 2003 3 2306 2311 12727854

[B109] Mani SA Guo W Liao M-J Eaton EN Ayyanan A Zhou AY Brooks M Reinhard F Zhang CC Shipitsin M Campbell LL Polyak K Brisken C Yang J Weinberg RA The epithelial-mesenchymal transition generates cells with properties of stem cells Cell 2008 3 704 715 18485877 10.1016/j.cell.2008.03.027 PMC2728032

[B110] Morel A-P Lièvre M Thomas C Hinkal G Ansieau S Puisieux A Generation of breast cancer stem cells through epithelial-mesenchymal transition PLoS One 2008 3 e2888 18682804 10.1371/journal.pone.0002888 PMC2492808

[B111] Li X Lewis MT Huang J Gutierrez C Osborne CK Wu M-F Hilsenbeck SG Pavlick A Zhang X Chamness GC Wong H Rosen J Chang JC Intrinsic resistance of tumorigenic breast cancer cells to chemotherapy J Natl Cancer Inst 2008 3 672 679 18445819 10.1093/jnci/djn123

[B112] Thomson S Buck E Petti F Griffin G Brown E Ramnarine N Iwata KK Gibson N Haley JD Epithelial to mesenchymal transition is a determinant of sensitivity of non–small-cell lung carcinoma cell lines and xenografts to epidermal growth factor receptor inhibition Cancer Res 2005 3 9455 9462 16230409 10.1158/0008-5472.CAN-05-1058

[B113] Thomson S Petti F Sujka-Kwok I Epstein D Haley J Kinase switching in mesenchymal-like non-small cell lung cancer lines contributes to EGFR inhibitor resistance through pathway redundancy Clin Exp Metastasis 2008 3 843 854 18696232 10.1007/s10585-008-9200-4

[B114] Byers LA Diao L Wang J Saintigny P Girard L Peyton M Shen L Fan Y Giri U Tumula PK Nilsson MB Gudikote J Tran H Cardnell RJG Bearss DJ Warner SL Foulks JM Kanner SB Gandhi V Krett N Rosen ST Kim ES Herbst RS Blumenschein GR Lee JJ Lippman SM Ang KK Mills GB Hong WK Weinstein JN An epithelial–mesenchymal transition gene signature predicts resistance to EGFR and PI3K inhibitors and identifies Axl as a therapeutic target for overcoming EGFR inhibitor resistance Clin Cancer Res 2013 3 279 290 23091115 10.1158/1078-0432.CCR-12-1558 PMC3567921

[B115] Barr S Thomson S Buck E Russo S Petti F Sujka-Kwok I Eyzaguirre A Gibson NW Miglarese M Epstein D Bypassing cellular EGF receptor dependence through epithelial-to-mesenchymal-like transitions Clin Exp Metastasis 2008 3 685 693 18236164 10.1007/s10585-007-9121-7 PMC2471394

[B116] Yi JM Dhir M Van Neste L Downing SR Jeschke J Glöckner SC de Freitas Calmon M Hooker CM Funes JM Boshoff C Genomic and epigenomic integration identifies a prognostic signature in colon cancer Clin Cancer Res 2011 3 1535 1545 21278247 10.1158/1078-0432.CCR-10-2509 PMC3077819

[B117] de Boer M van Dijck JA Bult P Borm GF Tjan-Heijnen VC Breast cancer prognosis and occult lymph node metastases, isolated tumor cells, and micrometastases J Natl Cancer Inst 2010 3 410 425 20190185 10.1093/jnci/djq008

[B118] Volinia S Galasso M Sana ME Wise TF Palatini J Huebner K Croce CM Breast cancer signatures for invasiveness and prognosis defined by deep sequencing of microRNA Proc Natl Acad Sci 2012 3 3024 3029 22315424 10.1073/pnas.1200010109 PMC3286983

[B119] Bastid J EMT in carcinoma progression and dissemination: facts, unanswered questions, and clinical considerations Cancer Metastasis Rev 2012 3 277 283 22215472 10.1007/s10555-011-9344-6

[B120] De Sousa E Melo F Wang X Jansen M Fessler E Trinh A de Rooij LPMH de Jong JH de Boer OJ van Leersum R Bijlsma MF Rodermond H van der Heijden M van Noesel CJM Tuynman JB Dekker E Markowetz F Medema JP Vermeulen L Poor-prognosis colon cancer is defined by a molecularly distinct subtype and develops from serrated precursor lesions Nat Med 2013 3 614 618 10.1038/nm.3174 23584090

[B121] Jie D Zhongmin Z Guoqing L Sheng L Yi Z Jing W Liang Z Positive expression of LSD1 and negative expression of E-cadherin correlate with metastasis and poor prognosis of colon cancer Dig Dis Sci 2013 3 1581 1589 23314859 10.1007/s10620-012-2552-2

[B122] Tobin NP Sims AH Lundgren KL Lehn S Landberg G Cyclin D1, Id1 and EMT in breast cancer BMC Cancer 2011 3 417 21955753 10.1186/1471-2407-11-417 PMC3192789

[B123] Lee J Yang G Paik S Chung M Does E-cadherin or N-cadherin or epithelial-mesenchymal transition have a probability of clinical implication of the prognostic marker in invasive ductal carcinoma? Cancer Res 2012 3 Abstract nr P2-10-39

[B124] Behnsawy HM Miyake H Harada K Fujisawa M Expression patterns of epithelial-mesenchymal transition markers in localized prostate cancer: significance in clinicopathological outcomes following radical prostatectomy BJU Int 2013 3 30 37 23107154 10.1111/j.1464-410X.2012.11551.x

[B125] Whiteland H Spencer-Harty S Thomas DH Davies C Morgan C Kynaston H Bose P Fenn N Lewis PD Bodger O Jenkins S Doak SH Putative prognostic epithelial-to-mesenchymal transition biomarkers for aggressive prostate cancer Exp Mol Pathol 2013 3 220 226 23933194 10.1016/j.yexmp.2013.07.010

[B126] Tanaka Y Terai Y Kawaguchi H Fujiwara S Yoo S Tsunetoh S Takai M Kanemura M Tanabe A Ohmichi M Prognostic impact of EMT (epithelial-mesenchymal-transition)-related protein expression in endometrial cancer Cancer Biol Ther 2013 3 13 23114646 10.4161/cbt.22625 PMC3566047

[B127] Rodrıguez-Gonzalez FG Mustafa DAM Mostert B Sieuwerts AM The challenge of gene expression profiling in heterogeneous clinical samples Methods 2013 3 47 58 22652627 10.1016/j.ymeth.2012.05.005

[B128] Alkatout I Wiedermann M Bauer M Wenners A Jonat W Klapper W Transcription factors associated with epithelial–mesenchymal transition and cancer stem cells in the tumor centre and margin of invasive breast cancer Exp Mol Pathol 2013 3 168 173 22985790 10.1016/j.yexmp.2012.09.003

[B129] Aoyagi K Tamaoki M Nishumura T Sasaki H Technical considerations for analyzing EMT–MET data from surgical samples Cancer Lett 2013 3 105 110 23933174 10.1016/j.canlet.2013.08.001

[B130] Yeung T Georges PC Flanagan LA Marg B Ortiz M Funaki M Zahir N Ming W Weaver V Janmey PA Effects of substrate stiffness on cell morphology, cytoskeletal structure, and adhesion Cell Motil Cytoskeleton 2005 3 24 34 15573414 10.1002/cm.20041

[B131] Nieto MA The ins and outs of the epithelial to mesenchymal transition in health and disease Annu Rev Cell Dev Biol 2011 3 347 376 21740232 10.1146/annurev-cellbio-092910-154036

[B132] Boyer B Vallés AM Edme N Induction and regulation of epithelial–mesenchymal transitions Biochem Pharmacol 2000 3 1091 1099 11007946 10.1016/s0006-2952(00)00427-5

[B133] Kalluri R Neilson EG Epithelial-mesenchymal transition and its implications for fibrosis J Clin Investig 2003 3 1776 1784 14679171 10.1172/JCI20530 PMC297008

[B134] Chen YL Lv J Ye XL Sun MY Xu Q Liu CH Min LH Li HP Liu P Ding X Sorafenib inhibits transforming growth factor beta1-mediated epithelial-mesenchymal transition and apoptosis in mouse hepatocytes Hepatology 2011 3 1708 1718 21360571 10.1002/hep.24254

[B135] Zeisberg M Hanai J-i Sugimoto H Mammoto T Charytan D Strutz F Kalluri R BMP-7 counteracts TGF-[beta] 1-induced epithelial-to-mesenchymal transition and reverses chronic renal injury Nat Med 2003 3 964 968 12808448 10.1038/nm888

[B136] Liu Y Epithelial to mesenchymal transition in renal fibrogenesis: pathologic significance, molecular mechanism, and therapeutic intervention J Am Soc Nephrol 2004 3 1 12 14694152 10.1097/01.asn.0000106015.29070.e7

[B137] Peláez-García A Barderas R Torres S Hernández-Varas P Teixidó J Bonilla F de Herreros AG Casal JI FGFR4 role in epithelial-mesenchymal transition and its therapeutic value in colorectal cancer PLoS One 2013 3 e63695 23696849 10.1371/journal.pone.0063695 PMC3655941

[B138] Zhang J Chen Y-L Ji G Fang W Gao Z Liu Y Wang J Ding X Gao F Sorafenib inhibits epithelial-mesenchymal transition through an epigenetic-based mechanism in human lung epithelial cells PLoS One 2013 3 e64954 23741434 10.1371/journal.pone.0064954 PMC3669213

[B139] Feldmann G Dhara S Fendrich V Bedja D Beaty R Mullendore M Karikari C Alvarez H Iacobuzio-Donahue C Jimeno A Gabrielson KL Matsui W Maitra A Blockade of hedgehog signaling inhibits pancreatic cancer invasion and metastases: a new paradigm for combination therapy in solid cancers Cancer Res 2007 3 2187 2196 17332349 10.1158/0008-5472.CAN-06-3281 PMC3073370

[B140] Feldmann G Fendrich V McGovern K Bedja D Bisht S Alvarez H Koorstra JB Habbe N Karikari C Mullendore M Gabrielson KL Sharma R Matsui W Maitra A An orally bioavailable small-molecule inhibitor of Hedgehog signaling inhibits tumor initiation and metastasis in pancreatic cancer Mol Cancer Ther 2008 3 2725 2735 18790753 10.1158/1535-7163.MCT-08-0573 PMC2605523

[B141] Fuchs BC Fujii T Dorfman JD Goodwin JM Zhu AX Lanuti M Tanabe KK Epithelial-to-mesenchymal transition and integrin-linked kinase mediate sensitivity to epidermal growth factor receptor inhibition in human hepatoma cells Cancer Res 2008 3 2391 2399 18381447 10.1158/0008-5472.CAN-07-2460

[B142] Choi Y-L Bocanegra M Kwon MJ Shin YK Nam SJ Yang J-H Kao J Godwin AK Pollack JR LYN is a mediator of epithelial-mesenchymal transition and a target of dasatinib in breast cancer Cancer Res 2010 3 2296 2306 20215510 10.1158/0008-5472.CAN-09-3141 PMC2869247

[B143] Menendez JA Metformin regulates breast cancer stem cell ontogeny by transcriptional regulation of the epithelial-mesenchymal transition (EMT) status Cell Cycle 2010 3 3807 3814 20890129

[B144] Shankar S Nall D Tang SN Meeker D Passarini J Sharma J Srivastava RK Resveratrol inhibits pancreatic cancer stem cell characteristics in human and KrasG12D transgenic mice by inhibiting pluripotency maintaining factors and epithelial-mesenchymal transition PLoS One 2011 3 e16530 21304978 10.1371/journal.pone.0016530 PMC3031576

[B145] Koorstra J Karikari CA Feldmann G Bisht S Rojas PL Offerhaus G Alvarez H Maitra A The Axl receptor tyrosine kinase confers an adverse prognostic influence in pancreatic cancer and represents a new therapeutic target Cancer Biol Ther 2009 3 618 626 19252414 10.4161/cbt.8.7.7923 PMC2678175

